# Bilateral thalamic lesions in a patient with probable acute disseminating encephalomyelitis: a case report

**DOI:** 10.1186/s12883-020-01834-w

**Published:** 2020-07-01

**Authors:** Yiming Zheng, Wei Zhang, Hongjun Hao, Feng Gao

**Affiliations:** Department of Neurology, Peking University First Hospital, No.8 Xishiku Street, Xicheng District, Beijing, 100034 China

**Keywords:** ADEM, Neurology image, Thalamic lesions

## Abstract

**Background:**

Bilateral thalamic lesions are rare. Here, we describe a case of probable acute disseminating encephalomyelitis (ADEM) with symmetrical bilateral thalamic lesions.

**Case presentation:**

An 85-year-old man presented with weakness of the lower limbs and urinary retention for 1 day, soon followed by coma. He had an H1N1 influenza vaccination 3 months ago. A lumbar puncture showed positive oligoclonal bands and negative results for anti-AQP4 antibodies. A head MRI demonstrated focal symmetrical bilateral thalamic lesions. An MRI of the thoracic spinal cord showed longitudinally extensive lesions in the spinal cord. He was diagnosed with probable ADEM. Despite being treated with IVIG, the patient remained unconscious and died a month later from pneumonia.

**Conclusions:**

In cases with bilateral thalamic lesions, the possibility of ADEM should be considered. The characteristics of the thalamic lesions and imaging findings in other parts of the brain or spinal cord should be taken into account in association with the clinical and laboratory information in making a correct diagnosis.

## Background

Bilateral thalamic lesions are rare. Both focal and systemic disorders may manifest as bithalamic abnormalities, including neoplastic, infectious, vascular, toxic, metabolic, and demyelinating disorders and disorders of congenital origin [1]. Here, we report a case of probable ADEM with symmetrical bilateral thalamic lesions.

## Case presentation

An 85-year-old man with a previous history of hypertension and diabetes mellitus presented with progressive weakness in the lower limbs, associated with urinary retention for 1 day. He did not have a fever. Neurological examination revealed paraplegia with no sensory disturbance, vision impairments or signs of meningeal irritation. He had delirium during the first night of hospitalization, followed by coma the next day. Except for an H1N1 influenza vaccination 3 months ago, there had been no preceding infections or other vaccinations. No recurrent oral ulcerations and urogenital lesions were found.

Extensive laboratory investigation showed an only slightly elevated white blood cell count (11.6 × 10^^9^ /L, Reference Range: 3.5–9.5), C-reactive protein level (10 mg/L, Reference Range: 0–8), and positive for antinuclear antibodies (1:1000, negative < 1:100). Serum sodium concentration, anti-dsDNA and ENA antibodies, ANCA and RF were all in the normal ranges. The tumor markers were unremarkable. A chest CT and abdominal ultrasound did not find evidence of cancer. Cerebrospinal fluid (CSF) examination showed normal intracranial pressure and his CSF was a crystalline fluid with 2 leukocytes/uL, containing 6.11 mmol/L of glucose (Reference Range: 2.5–4.5). His CSF contained 0.63 g/L of protein (Reference Range: 0.15–0.45). The IgG index of the CSF was elevated at 4.13 (Reference Range: < 0.85). Oligoclonal bands (Type III) (HYDRAGEL 3 & 9 ISOFOCUSING gel) were found in the CSF. Antibodies in the CSF for cytomegalovirus, Epstein-Barr virus and herpes simplex virus were all negative. No infectious pathogens were identified in the blood or in the CSF. Anti-Hu, anti-Yo, anti-Ri, anti-Amfi, anti-CV2, anti-Ma2, anti-NMDAR, anti-VGKC, anti-AMPAR, anti-GABA_B,_ anti-AQP4 and anti-MOG antibodies in the blood and CSF were all negative.

**Fig. 1:**
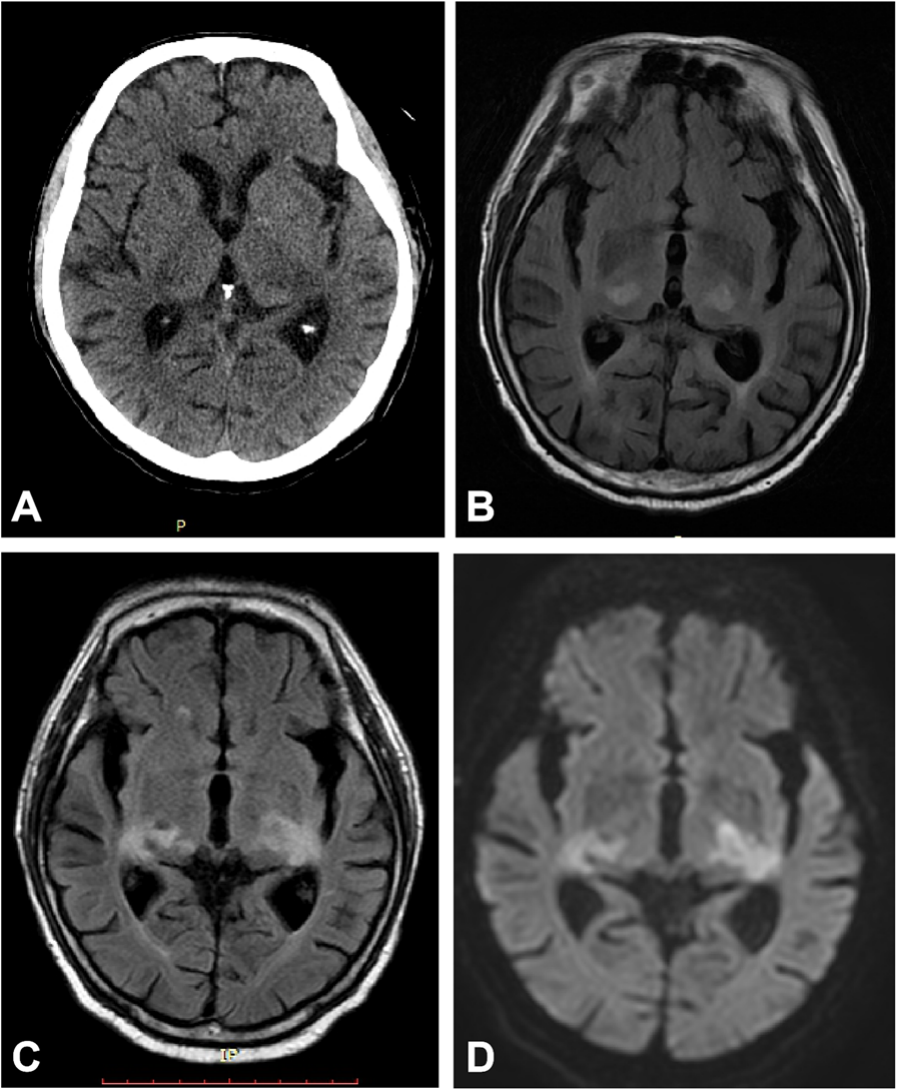
CT and MRI scans showing bilateral thalamic lesions. The CT scan of the head shows low density changes in the bilateral thalamic lesions (**a**). Axial FLAIR image reveals symmetric hyperintense signal alterations in thalami (**b**) and enlargement of the lesions after 17 days (**c**) with mild hyperintensity changes in DWI (**d**)

Magnetic resonance imaging (MRI) of the brain on the night of hospitalization demonstrated focal symmetrical bilateral lesions in the thalamus with hyperintense T2 and fluid-attenuated inversion recovery (FLAIR). A head MRI after 17 days showed enlargement of the bilateral thalamic lesions with low density changes in computed tomography. The diffusion-weighted images (DWI) showed a slightly high intensity (Fig. 1). No evidence was found of signal changes in the deep cerebral veins or the straight sinus. There were also progressive multiple lesions in the subcortical white matter, brain stem and a hyperintense long segment of T2 in the thoracic spinal cord. The cranio-cervical computerized tomography angiography (CTA) was unremarkable. (Supplementary materials).

Given the advanced age of this diabetic patient, we did not offer treatment with intravenous methylprednisolone. Despite treatment with IV immunoglobulin (IVIG) (0.4 g/kg/d, for 5 days), the patient remained unconscious. He developed pneumonia and passed away 38 days after onset from respiratory failure.

## Discussion and conclusions

Our patient had a polyfocal clinical CNS event following an influenza vaccination. A review of 15 cases reported as either encephalomyelitis or ADEM following influenza vaccination published since 1982 revealed that neurological symptoms typically developed within 3 weeks of vaccination and patients generally made a good recovery [2]. This patient was, however, older than the others and had significant brainstem involvement, which may be the causes of the poor prognosis [3]. Although there have been a few cases in which clinical presentation has been delayed even up to 4–5 months after HPV immunization [4], there is still no clear answer as to whether the H1N1 influenza vaccination 3 months before onset was the cause of demyelination in this case.

There are many points in favor of the diagnosis of ADEM, including rapid deterioration, features of encephalopathy, multifocal neurologic symptoms and brain MRI abnormalities consistent with demyelination. However, ADEM remains a diagnosis of exclusion [5]. No other etiology has been found to explain these events in this patient. One important differential diagnosis for this patient is central nervous system infection, especially viral encephalitis or meningoencephalitis. As we know, Japanese patients with encephalitis may present with bilateral thalamic lesions [1]. However, this patient became sick in the winter, and had no history of fever before onset, presented with not only encephalopathy but also myelopathy, and exhibited no signs of meningism. The CSF test showed normal intracranial pressure, leukocyte count and negative results for some common virus antibodies. Other autoimmune antibody-mediated diseases were excluded by the negative results of the antibody tests including anti-AQP4, anti-MOG and many other antibodies related to paraneoplastic syndrome. The tests, including the chest CT, abdominal ultrasound, tumor markers and paraneoplastic antibodies, were all negative. However, a whole-body PET-CT would have been needed to rule out paraneoplastic processes considering the patient’s age. Significant diffusion restrictions due to high cell density is often suggestive of a diagnosis of primary CNS lymphoma [6]. However, the DWI images of this patient showed a slightly high intensity which can be seen in the acute phase of ADEM [3]. It was a pity that this patient did not have MRI contrast enhancement because of the worry over the side effects of the contrast medium after a CTA test in this aged patient. Nonetheless, imaging alone does not allow CNS lymphoma to be discriminated from other malignant tumors or inflammatory lesions. In primary isolated CNS angitis, images on MRI scans of the brain typically show deep brain infarcts, which were absent in our patient. Furthermore, his CT angiography was unremarkable. The rapid progress over a few days and the longitudinally extensive lesions in the thoracic spinal cord did not support a diagnosis of lymphoma or vasculitis. However, lymphoma or vasculitis could only have been totally excluded by a biopsy [6]. Patients with neuro-Behçet disease can presented as ADEM-like manifestations. However, this patient had no recurrent oral ulcerations and urogenital lesions [7].

Most patients with bilateral thalamic lesions have characteristic imaging findings to narrow the differential diagnosis. In addition to thalamic findings and imaging findings for other parts of the brain or spinal cord should be taken into account in association with the clinical and laboratory information in making a correct diagnosis [1, 8, 9]. The lesions in ADEM typically involve the subcortical and central white matter and cortical gray-white matter junction, thalami, basal ganglia, cerebellum and brainstem [9]. Bilateral thalamic lesions have been reported in 12% of pediatric patients with ADEM [10]. However, the pattern of thalamic lesions in ADEM can be variable. Bilateral symmetric parts of the thalamus were most affected in this patient, which has not been reported in the previous literature as far as we know [1, 10, 11]. The involvement pattern in this patient was different from other pathology affecting certain other parts of the thalamus, including paramedian involvement for infarction of the Percheron artery, medial involvement for Wernicke encephalopathy, or ventrolateral involvement for Wilson’s disease. However, bilateral involvement of the entire thalamus can be also seen in ADEM, similar to bithalamic glioma, flavivirus infection and deep vein thrombosis [1, 8, 9]. No evidence of signal changes in the deep cerebral veins or the straight sinus was found. In deep vein thrombosis, bilateral involvement of the entire thalamus is affected, which was not the case with our patient. Furthermore, the involvements of white matter and the spinal cord were helpful in the diagnosis of ADEM in our patient [5]. Many lesions, including the bilateral thalamic lesions in this patient, become enlarged after a few days, which is common in ADEM [12]. Therefore, we consider the diagnosis for this patient is probable ADEM with bilateral thalamic lesions.

In conclusion, the possibility of ADEM should be considered when bilateral thalamic lesions have been detected. The characteristics of thalamic lesions and imaging findings in other parts of the brain or spinal cord should be taken into account in association with the clinical and laboratory information in making a correct diagnosis. The diagnosis for our patient is probable ADEM. We could not totally exclude other diagnoses.

## Supplementary information

**Additional file 1.**

## Data Availability

All data related to this case report are documented within this manuscript.

## Abbreviations

ADEM: Acute disseminating encephalomyelitis
MRI: Magnetic resonance imaging
FLAIR: Fluid attenuated inversion recovery
ENA: Extracted nuclear antigens
ANCA: Antineutrophil cytoplasmic antibody
RF: Rheumatoid factor
CSF: Cerebrospinal fluid
NMDAR: N-methyl-D-aspartic acid receptor
VGKC: Voltage-gated potassium channel
AMPAR: Aminomethyl phosphoric acid receptor
GABA_B_: Gamma-amino butyric acid
AQP4: Aquaporin 4
MOG: Myelin oligodendrocyte glycoprotein
