# Supplementary material for: Bilateral thalamic lesions in a patient with probable acute disseminating encephalomyelitis: a case report
Source: BMC Neurol. 2020 Jul 1;20:265. doi: 10.1186/s12883-020-01834-w (PMC7328267; doi:10.1186/s12883-020-01834-w)
Supplement: Supplementary file 1 — Additional file 1. [file 12883_2020_1834_MOESM1_ESM.doc]

**Supplementary materials:**

**eFigure 1. MRI of the thoracic spinal cord**


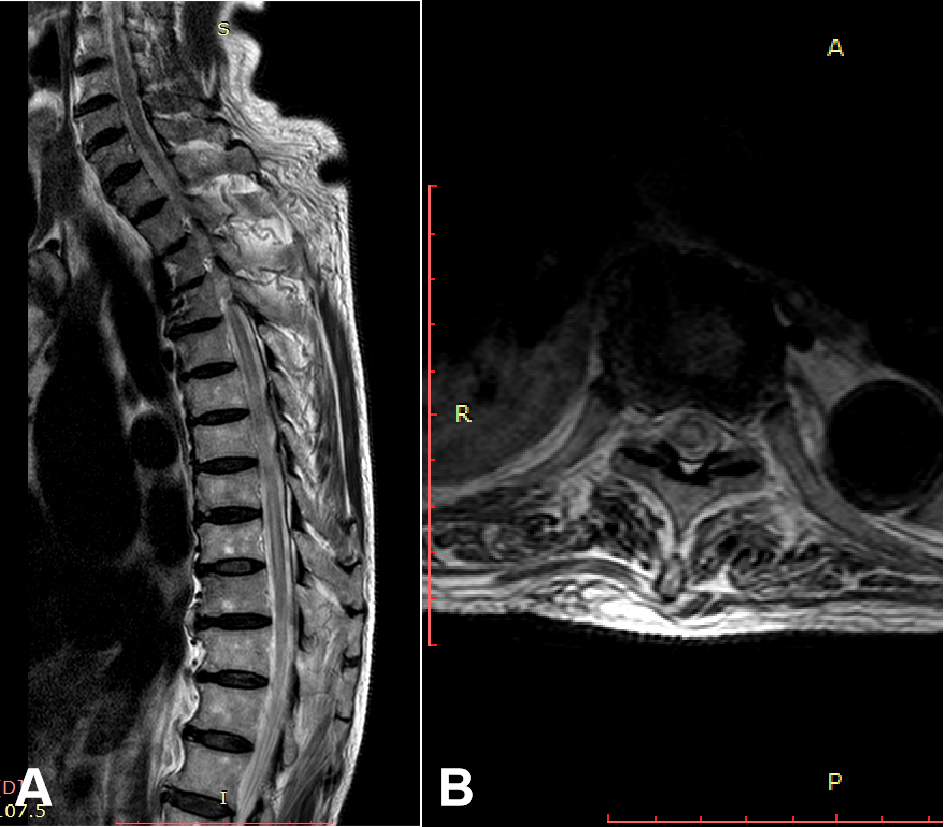
Sagittal T2-weighted image of spinal cord (A) demonstrates focal areas of high signal in the thoracic cord. Axial T2-weighted image (B) shows abnormal signal intensities in spinal cord.

**eFigure 2. MRI of the head showing progressive multiple lesions**


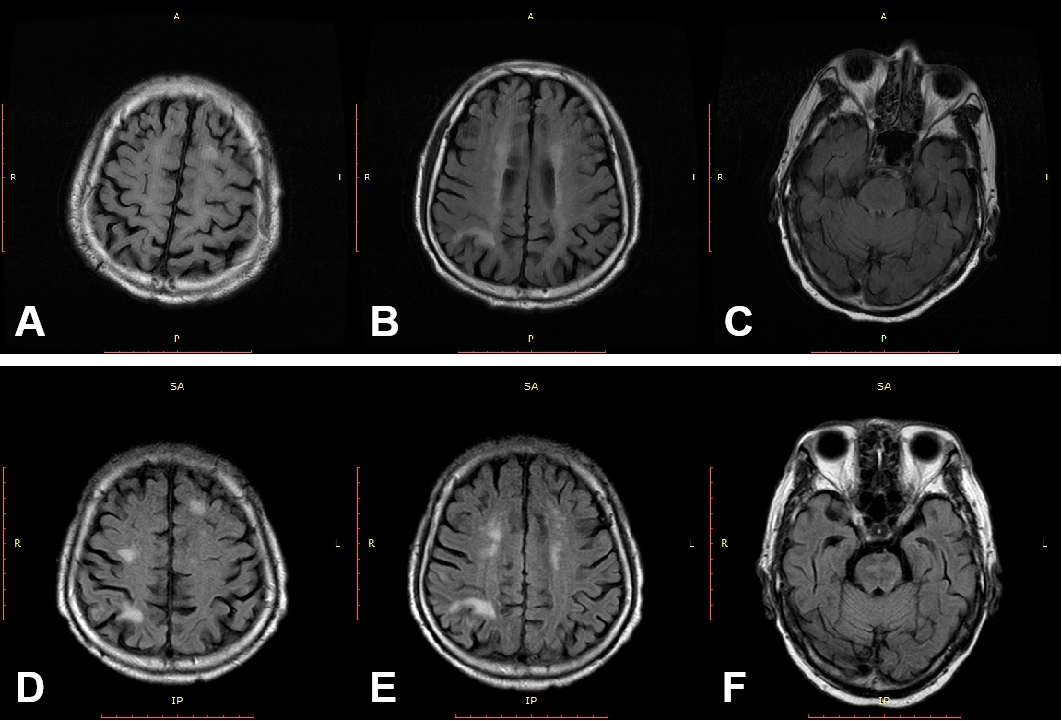


Axial FLAIR images showing progressive multiple lesions in subcortical white matter (A,B the first MRI and D,E 17 days after), and brain stem (C, the first MRI and F, 17 days after).

**eFigure 3. The cranio-cervical computerized tomography angiography (CTA)**


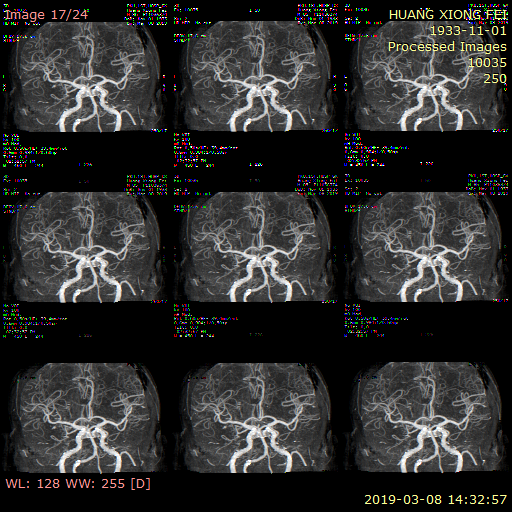


No obvious vascular stenosis was found in the CTA test.
